# Nucleic Acid Vaccines Encoding Proteins and Virus-like Particles for HIV Prevention

**DOI:** 10.3390/vaccines12030298

**Published:** 2024-03-12

**Authors:** Ferran Tarrés-Freixas, Bonaventura Clotet, Jorge Carrillo, Julià Blanco

**Affiliations:** 1 IrsiCaixa AIDS Research Institute, Crta del Canyet S/N, 08916 Badalona, Spain; 2 Centre for Health and Social Care Research (CESS), Faculty of Medicine, University of Vic-Central University of Catalonia (UVic-UCC), 08500 Vic, Spain; 3 Infectious Diseases Department, Germans Trias i Pujol Hospital, 08916 Badalona, Spain; 4 CIBER de Enfermedades Infecciosas, ISCIII, 28029 Madrid, Spain; 5 Germans Trias i Pujol Research Institute (IGTP), 08916 Badalona, Spain

**Keywords:** AIDS, mRNA, DNA, VLP, antibody, bNAbs, Env, Gag

## Abstract

The development of HIV prophylactic vaccines is facing an impasse, since all phase IIb/III clinical trials were halted in 2023 without demonstrating efficacy. Thus, the field is in need of developing novel immunogens and vaccination strategies that induce broadly neutralising antibodies together with potent Fc-dependent effector functions, as well as protective cross-reactive CD4^+^ and CD8^+^ T cell responses. Nucleic acid vaccines, particularly mRNA vaccines, have been one of the major groundbreaking advances in the current decade. Nucleic acid vaccines may help recalibrate the HIV vaccine field towards the use of delivery systems that allow the proper expression of immunogens as a sole antigen (i.e., membrane-bound trimeric envelope glycoproteins) or even to be displayed in a multiantigen platform that will be synthesised by the host. In this review, we will summarise how the multiple HIV vaccine strategies pursued in the last 40 years of HIV research have driven current vaccine development, which are the most relevant immunogens identified so far to induce balanced adaptive immune responses, and how they can benefit from the acceptance of nucleic acid vaccines in the market by reducing the limitations of previous delivery systems. The incorporation of nucleic acid vaccines into the current heterogeneous repertoire of vaccine platforms may represent an invaluable opportunity to reignite the fight against HIV.

## 1. Introduction

Vaccines are indisputably one of the most successful achievements in the biomedical field. Vaccination programmes have been pivotal to extending life expectancy in the last century [1,2]. Yet, infectious diseases represent a continuous threat to society. For instance, estimates indicate that there is a 2% chance of new pathogens emerging in any given year [3]. And while emerging pathogens can mould history, as recently demonstrated by the Severe Acute Respiratory Syndrome Coronavirus 2 (SARS-CoV-2) pandemic or previously by the Human Immunodeficiency Virus (HIV), so can vaccines. And indeed, nucleic acid vaccines were game changers during the SARS-CoV-2 pandemic. Both the adaptation of classical vaccine platforms and the approval of novel technologies yielded several candidates that lowered the disease burden of the Coronavirus Disease 2019 (COVID-19) [4]. 

The SARS-CoV-2 pandemic experienced an important achievement with the approval of the first nucleic acid-based vaccines for human use, either as deoxyribnonucleic acid (DNA) vaccines [5], or messenger ribonucleic acid (mRNA) vaccines [6,7]. mRNA vaccines proved to be especially efficacious and competitive, and they are considered as one of the vaccine platforms that might achieve the highest growth in the near future, even beyond the infectious disease field [8,9]. However, research on nucleic acid vaccines predates COVID-19, and these types of vaccines were already being developed for HIV. 

In contrast to the sharp success of SARS-CoV-2 vaccines, forty years of vaccine research against HIV have only yielded modest successes in efficacy trials (i.e., RV144) [10] or no success at all [11,12,13,14,15]. While it is true that SARS-CoV-2 vaccine development benefited from many years of scientific advances in both coronaviruses and other fields (e.g., gene delivery, mRNA engineering, and HIV vaccine research) [16,17,18,19,20], it is still remarkable that vaccines with such a high efficacy could move from bench to market within such a short time. The introduction of SARS-CoV-2 nucleic acid vaccines into the market, have shown that mRNA vaccine technology is a powerful tool for speeding up the development of novel vaccines in several areas including the HIV vaccine field.

In this review, we will provide a summary of the different HIV vaccine strategies that have been tested from the beginning of the acquired immunodeficiency syndrome (AIDS) pandemic, with a particular focus on the selected immunogens and vaccine platforms. In addition, we will discuss how HIV vaccine field can benefit from the different advantages of nucleic acid vaccines. 

## 2. Which Immunogens Should We Use and Why?

HIV immunogens have been classically categorised into two main groups: those designed to elicit protective humoral responses, mainly neutralizing antibodies (NAbs), and those devised to induce potent cellular immune responses associated with the control of disease progression [21,22]. 

### 2.1. Elicitation of Protective Humoral Responses

HIV envelope glycoprotein (Env) is the only viral protein present on the viral surface, and it is the target of NAbs [23,24]. It is a highly glycosylated heterotrimeric protein generated by the oligomerisation of three gp160 molecules. Each gp160 monomer contains two subunits (gp120 and gp41) that are responsible for CD4 recognition and membrane fusion, respectively [25]. However, Env induces poorly protective CD4^+^ and CD8^+^ T cell responses [26].

**Figure 1:**
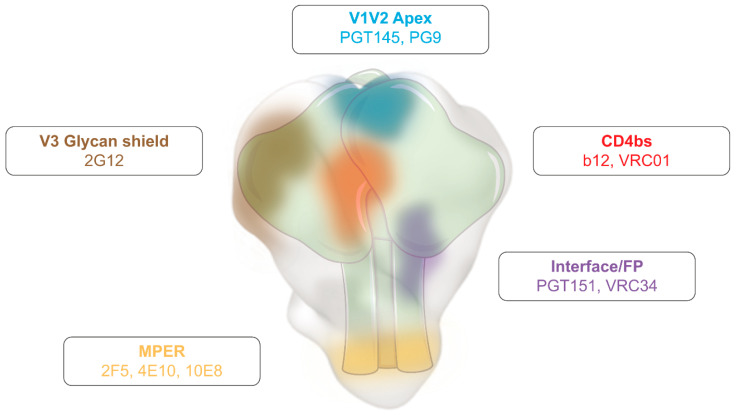
Human immunodeficiency virus (HIV) Envelope glycoprotein (Env) vulnerability map. Schematic representation of Env, highlighting the main vulnerability domains and examples of broadly neutralising antibodies (bNAbs) that target these regions. Adapted from [28].

Env accumulates the highest degree of genetic variability of HIV, which can be as high as 40% across HIV subtypes [27]. Despite this, Env has several conserved domains that play a major role in protein architecture and function (Figure 1), such as the CD4 binding site (CD4bs) in gp120, or the fusion peptide (FP) and the Membrane Proximal External Region (MPER) in the gp41 subunit [23,28,29,30]. These functional domains are known as HIV vulnerability sites since they are targeted by NAbs. However, owing to HIV’s exceptional mutational rate, HIV can evade the immune pressure exerted by these NAbs and escape their effect [31]. Furthermore, stemming from HIV’s enormous genetic diversity, NAb sensitivity varies among different HIV clades or subtypes. 

Despite the enormous antigenic variability of Env and the rapid virus/immune response adaptation, broadly neutralizing antibodies (bNAbs) that block multiple HIV subtypes showed in vivo antiviral activity in humans [32,33,34]. bNabs are only elicited in 1% of people living with HIV (PLWH) [35], and target several of Env’s vulnerability domains (e.g., CD4bs [b12 and VRC01], MPER [2F5, 10E8], among others) (Figure 1). They are the result of a long and complex antigen selection process, which explains part of their main features, such as the high degree of somatic hypermutation and the long time to elicitation after infection [36]. Still, their detection in humans is proof that they can be elicited by a proper stimulation [37]. 

bNAbs can cross-neutralise a broad range of HIV variants, and protect from HIV acquisition, particularly if a combination of bNAbs that target different vulnerability regions are co-administered [38,39,40]. Therefore, their elicitation by vaccination may be pivotal for the success of any prophylactic HIV vaccine [29]. 

Strong efforts have been taken for the design of Env-based immunogens that can elicit bNAbs. In an initial attempt, Env glycoproteins and subunit vaccines were tested in multiple forms (Gp160/gp140, gp120 [AIDSVAX B/B or B/E], or gp41) with limited success [14,15,41,42,43,44]. These strategies were unable to induce broadly protective responses, and the virus was able to evade them [31]. Despite the fact that these initial immunogens failed at eliciting NAbs, more advanced Env-based immunogens have been developed in the last few years. Some examples of these rationally designed HIV immunogens are: (i) eOD-GT8, that displays CD4bs antigens to prime germline precursors of known bNAbs [45]; (ii) linear epitope-based vaccines such as the fusion peptide (FP) or the MPER, designed to reduce the generation of non-neutralising antibodies [30,46]; or (iii) stable trimeric Env immunogens that maintain the prefusion native conformation of Env and are mainly targeted by Nabs, which may be SOSIP trimers [47,48,49], native flexible linked (NFL) trimers [50], or uncleaved prefusion optimised (UFO) trimers [51]. Although these immunogens can elicit cross-neutralising responses, they need the coadministration of adjuvants to increase their immunogenicity, particularly when they are assayed as recombinant proteins [e.g., alum phosphate [15,43,52], squalene-based adjuvants [53,54,55], or complex liposomes [56,57]]. 

In addition to neutralising humoral responses, antibody-dependent cellular responses (like antibody-dependent cellular cytotoxicity [ADCC] or antibody-dependent cell phagocytosis [ADCP]) can also play a pivotal role in providing certain degree of protection against HIV, as demonstrated by the RV144 vaccine Thai trial [58,59,60]. Importantly, these functions are mainly mediated by the constant region (Fc) of IgG1 and IgG3 immunoglobulins, whose production after antibody class-switch recombination is modulated by the cytokine milieu [61] Since vaccine properties and the adjuvant used may influence this milieu, both factors are also determinant to elicitation of optimal humoral responses.

### 2.2. Elicitation of Protective T Cell Responses

Conversely to Env, the structural polyprotein composed by the group-specific antigen (Gag) and polymerase (Pol) Gag-Pol, and the “negative factor” (Nef) accessory protein can strongly stimulate T cell responses [62]. Anti-Gag-Pol antibodies can be elicited during HIV infection or vaccination, but they are non-neutralising since these proteins are located inside the viral particle. That is why they their administration as subunit proteins is not useful as a vaccination strategy. Still, they remain as useful diagnostic tools [63]. 

Gag, Pol and Nef have several conserved domains that can be exploited to generate broad and protective T cell responses [64]. In fact, CD4^+^ T helper responses against Gag have been correlated with a slower progression to AIDS [65,66]. Additionally, some CD8^+^ T cell responses may play a major role in controlling HIV since a few HLA-B alleles have been associated with long-term non-progressor and elite controller patient profiles [67,68], highlighting their potential as vaccine immunogens. 

Immunization studies have tested Gag-Pol and Nef immunogens as nucleic acid vaccines (as plasmid DNA or through viral vectors) [69,70,71]. The most used viral vectors include poxviruses [e.g., vaccinia viruses (MVA or NYVAC)] [72], canarypox viruses (e.g., ALVAC) [10], and adenoviral vectors (Ad5) [73]. Some examples of Gag-based immunogens administered as vaccines are: (i) mosaic proteins, that were bioinformatically designed to cover most HIV group M variants [74]; (ii) HIV.consv constructs, that encoded for the most conserved Gag elements [75]; or (iii) HTI immunogen, that contained conserved peptides that are targeted by effective cellular immune responses [76]. Nef has been included together with Gag and Pol in many of vaccine strategies (e.g., STEP, Phambili, HVTN505, PrEPVacc) [77]. These types of vaccines have been extensively evaluated for their capability to prime or boost cellular responses designed to kill infected cells, but they may also be relevant for prophylactic vaccine strategies [78]. 

Overall, considering the properties of each group of immunogens an optimal HIV vaccine strategy should induce a balanced adaptive immune response in which both protective humoral and cellular immune responses are elicited [21].

## 3. HIV Vaccine Efficacy Trials: Past Failures, Future Successes

**Figure 2:**
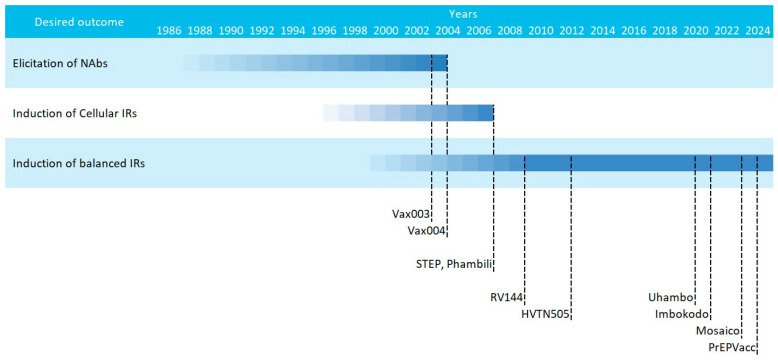
Scheme of the multiple HIV vaccine clinical trials classified by strategy. Starting dates are gradual and ending dates represent the publication year.

The current consensus that HIV vaccines should aim at inducing potent and balanced humoral and cellular responses was coined after the underperformance of multiple phase IIb/III HIV vaccine efficacy trials in the early 2000s. These attempts can be classified in three main waves, that followed different strategies [21,79]: (i) an early phase in which immunogens based on recombinant Env proteins were tested for the induction of humoral responses (Vax003 and Vax004); (ii) a second phase that saw the introduction of viral vectored vaccines that coded for HIV genes designed to induce cellular responses (STEP and Phambili); and (iii) a later phase in which vaccines were designed for inducing balanced humoral and cellular responses (RV144, Mosaico, PrEPVacc, etc.) (Figure 2). 

Despite these different strategies, researchers agreed from the beginning that the main issue with HIV vaccines would be the enormous global diversity of HIV. HIV group M is the main group (represents 90% of HIV infections) and encompasses multiple subtypes (A, B, C, D, F, G, H, J, and K) and circulating recombinant forms (CRF) [80]. Intragroup variability within a subtype can be as high as 15%, while it can reach up to 40% genetic variability between different subtypes [27]. Hence, two strategies were foreseen: either vaccines were designed to induce a broad response covering all subtypes and variants, a feat we have not yet achieved, or vaccines should be tailored to the predominant subtype in a specific geographical region. 

That was the situation with the two initial phase III trials (Vax003 and Vax004) that concluded in the year 2000, in which the gp120 subunit vaccine AIDSVAX was adapted to match predominant subtypes in Thailand (subtypes B/E) or in developed countries (subtype B) (clinicaltrials.gov: NCT00002441 and NCT00006327, respectively) [43]. These trials also differed on the target population; while Vax003 in Thailand targeted drug users, Vax004 in USA and the Netherlands was aimed at men who have sex with men (MSM) (Table 1). Still, although the antibody responses induced by these vaccines were robust, their cross-neutralising capacity was very low, and none of them showed overall efficacy, even if some groups showed non-significant trends of protection [81]. 

Once Vax003 and Vax004 ended, two phase IIb trials aimed at inducing potent T cell responses were being initiated and led by Merck (STEP study/HVTN502 and Phambili study/HVTN503) (Table 1). These trials rested on the fact that neutralising antibodies were not correlating with HIV disease progression, but cellular responses against Gag, Pol and Nef were [82]. Subtype B *gag*, *pol*, and *nef* were DNA encoded and delivered with adenoviral vectors (MRK Ad5). Despite testing the vaccine in two different settings with different prevalent subtypes (USA and Australia for STEP and South Africa for Phambili), vaccines were not adapted to the circulating South-African strain. In any case, both studies were halted in late 2007, not even a year after they started, since researchers found a higher incidence in the vaccinated group than the placebo one [70]. Further analyses indicated an association between higher HIV incidence and pre-existing anti-Ad5 antibodies, highlighting one of the main limitations of viral vector vaccines: the generation of anti-vector immune responses [11,83], a problem that can be bypassed with plasmidic DNA or mRNA vaccines.

Parallel to STEP and Phambili trials, a more successful phase III attempt was carried out. The RV144 (clinicaltrials.gov: NCT00223080) was designed to stimulate both arms of the adaptive immune system. The vaccination regimen included subtype B Gag-Pol and membrane-bound subtype AE Env immunogens delivered with a canarypox vector (vCP1521) named ALVAC-HIV and administered in four doses. The last two doses were co-administered with AIDSVAX B/E to boost humoral responses [44]. Despite some discussion on whether the foundation for this clinical trial were solid enough [84,85], the truth is that this is the only HIV vaccine efficacy trial to provide a positive but moderate degree of protection (31.2% protection of vaccinees over placebos) [10]. Deeper analyses highlighted the role of ADCC-mediating V1V2 humoral responses as correlates of protection [59], while anti-Env IgAs were associated with increased risk of HIV infection [58]. However, these results were considered too poor to merit further commercial development [86]. 

After the relative success of the RV144 Thai trial, all developed efficacy trials focused on the elicitation of balanced adaptive responses, using a similar protocol (HVTN702/Uhambo) or opening new avenues (HVTN505 and the HVTN705/Imbokodo-HVTN706/Mosaico trials). However, none of these trials achieved a similar success to the one reported with RV144. 

In chronological order, HVTN505 started in 2009, and tested for the first time in a phase IIb trial a DNA vaccine (clinicaltrials.gov: NCT00865566). In this trial, vaccinees were primed with six plasmids encoding for subtype B *gag*, *pol*, *nef*, subtype A, B and C *env* and boosted with a mix of four Ad5 coding for subtype B *gag-pol* and subtype A, B and C *env*. Unfortunately, this trial was stopped due to futility [87], and only modest anti-Env CD8^+^ cytotoxic responses were enhanced in vaccinated uninfected volunteers [88]. 

The HVTN702/Uhambo phase IIb/III clinical trial (clinicaltrials.gov: NCT02968849) tried to reproduce the RV144 success in South Africa by adapting the vaccine components to a subtype C variant (Table 1) [89], but it was stopped due to futility [54].

Another HIV vaccine strategy that was tested in efficacy trials was the mosaic constructs [90,91]. Two efficacy trials were performed in parallel using the same strategy, but the booster was adapted to the target population: HVTN705/Imbokodo focused on women from sub-Saharan Africa, while HVTN706/Mosaico targeted MSM from America and Europe (Table 1). The vaccination regimen was similar to RV144 and included priming with four doses of four viral vectors (Ad26) that encoded four mosaic constructs: Mos1.Gag-pol, Mos2.Gag-pol, Mos1.Env, and Mos2S.Env. Boosting was performed with a soluble protein (subtype C gp140 trimer for Imbokodo and a mosaic gp120 for Mosaico) in parallel with the third and fourth doses (clinicaltrials.gov: NCT03060629 and NCT03964415). Imbokodo was stopped in summer 2021 for futility, since it showed a non-significant trend of protection among vaccinees (25.2%) [92]. Mosaico continued until early 2023, when it was also stopped for futility [13]. 

The most recent addition to this lengthy list of halted clinical trials is PrEPVacc (clinicaltrials.gov: NCT04066881). In this phase IIb study performed in Uganda, two vaccinee groups were compared to placebo control, but all volunteers received pre-exposure prophylaxis (PrEP) treatment [77]. The main vaccine component was a DNA vaccine (DNA-HIV-PT123) encoding for subtype C *gag*, *env* and *pol-nef* [53]. The two vaccinated arms received either AIDSVAX on weeks 0, 4, 24 and 48 or subtype C gp140 (weeks 0 and 4) and subtype C gp140 with the MVA vector on weeks 24 and 48 [55]. The vaccine arm of this trial was stopped by recommendation of the independent data monitoring committee in December 2023 due to the absence of vaccine efficacy observed in the interim analysis [93]. 

As of January 2024, there are no active candidates in efficacy trials, despite a few examples of mRNA vaccines in phase I trials [94,95]. Furthermore, considering the lack of success in the previous trials, reticence may arise among stakeholders to continue pursuing HIV vaccines without a solid lead candidate, as discussed 20 years ago when the RV144 was initialising [84,85]. However, the breakthrough of mRNA vaccine platforms may turn the tables in this sense and reignite the field after reaching this critical impasse. Considering the contribution of the HIV vaccine field to our understanding of protective mechanisms of SARS-CoV-2 vaccines, it seems appropriate that the same field should now seize the opportunities opened by these novel mRNA vaccines [96]. The main advantages provided by mRNA vaccines and their relevance in HIV vaccine development will be discussed in the following sections. 

## 4. Nucleic Acid Vaccines and Their Delivery

Nucleic acid vaccines can be categorized into three groups: viral vectored vaccines, DNA vaccines, and mRNA vaccines. DNA- or RNA-encoded products are not novel in the biomedical field, since DNA or RNA viral vectors have been widely used in several applications ranging from gene therapy to vaccinology. For instance, viral vectors share many advantages with plasmid DNA or mRNA platforms, like a straightforward production protocol or a versatility to adapt the proteins they encode on a nucleic acid level, instead of having to produce, purify, and validate each product [97]. Furthermore, their protocols are well-optimised and standardised worldwide, facilitating technology exchanges and fast adaptation to new threats and challenges [98]. 

**Figure 3:**
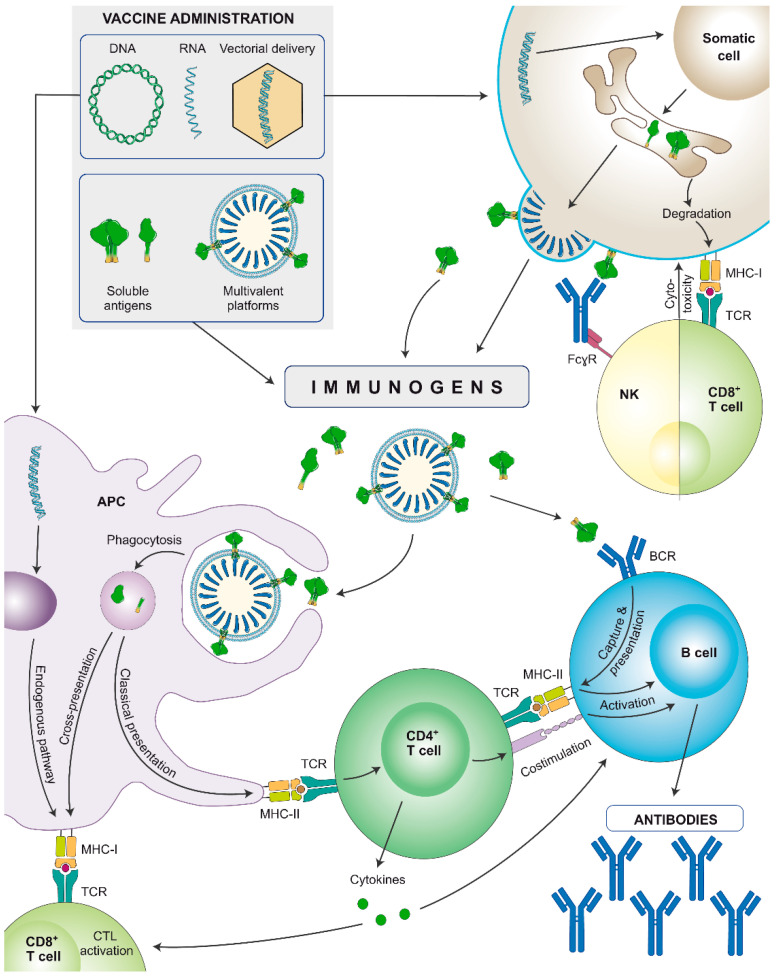
Schematic representation of the immune stimulation pathways of nucleic acid-encoded or soluble recombinant protein-based vaccines (subunit proteins or multivalent platforms). Nucleic acids are internalised by somatic cells that synthesize the immunogens. They can be secreted or expressed on the cell surface, depending on the immunogen design. Secreted immunogens will be recognised by professional antigen-presenting cells (APCs: B cells, dendritic cells, and/or macrophages), triggering an adaptive immune response. Additionally, APCs also internalise DNA and/or mRNA and may be transfected by viral vectors to produce the antigen, that will be presented through major histocompatibility complex (MHC)-I molecules, stimulating cellular responses. Cells: innate APC (purple), somatic cell (brown), NK cell (yellow), CD8^+^ T lymphocyte (light green), CD4^+^ T lymphocyte (dark green), B lymphocyte (blue). Proteins: antibodies (blue), monomeric/trimeric Env (green).

Nucleic acid-encoded vaccines are administered intramuscularly but instead of providing a direct stimulation to the immune system, they first need to be internalised by somatic cells that will synthesize the encoded antigens (Figure 3). They can encode soluble proteins that, once secreted, can stimulate the immune system in a similar fashion as subunit proteins, but additionally, during synthesis, they will be also processed by the proteosome and presented to CD8^+^ cytotoxic lymphocytes via major histocompatibility complex (MHC)-I. Alternatively, immunogens containing the transmembrane domain can be encoded and synthesized as membrane-anchored antigens, or multimeric presentations can be designed (as virus-like particles [VLPs]). In both cases aiming to better recruit B cell precursors and lower the affinity threshold [99]. As a case in point, Comirnaty (BNT162b2) and Spikevax (mRNA-1273) mRNA vaccines encode membrane-bound SARS-CoV-2 Spikes [100]. 

Furthermore, DNA and/or mRNA can be internalised by antigen-presenting cells (APCs), and the encoded proteins will be synthesized, processed, and presented via MHC to activate cellular responses. Overall, both nucleic acid-encoded and subunit vaccines induce strong humoral responses, but the former will better stimulate the cellular arm, and hence, induce a more balanced immune response [101,102]. Still, adjuvants can further help by polarising and potentiating the immune response induced by nucleic acid vaccines. 

### 4.1. Viral Vectored HIV Vaccines

There are several examples of viral vectored HIV vaccines tested in efficacy trials, as previously discussed, and viral vectors approved for use in humans (e.g., the canarypox vector ALVAC-HIV, vaccinia viruses [NYVAC, MVA, Tiantan], different adenoviruses [Ad5, Ad26, Ad35, chimpanzee adenovirus]) [72,103,104,105]. Their utility is indisputable, but there are some limitations that should be highlighted, such as pre-existing anti-vector responses that can interfere with the immunogenicity of the delivered antigen, as shown in the STEP study [11,106], or their elicitation upon vaccination. Considering that an HIV vaccine may require the administration of multiple doses [107], the issue with anti-vector responses is no minor concern, since it can reduce the effectivity of homologous vaccine regimens [108,109]. Furthermore, the potential of replication-competent vectors to reactivate is especially concerning in immunocompromised vaccinees, such as PLWH [110]. Even though there are many research lines with viral vectors trying to solve these matters (e.g., replication deficient vectors and combination of different serotypes and heterologous regimens), plasmid DNA and mRNA vaccines are an attractive alternative to bypass these limitations (Box 1) [111]. For example, SARS-CoV-2 mRNA vaccines have demonstrated efficacy in swiftly adjusting to emergent variants capable of evading prior immune responses. This characteristic of mRNA technology holds significant promise for HIV vaccine development, given the challenge posed by HIV’s capacity to circumvent immune defences, due to its exceptionally elevated mutational rate [112]. 

### 4.2. DNA and mRNA HIV Vaccines

DNA and mRNA vaccines have been extensively pursued in the last decades, both in preclinical and early phase clinical trials. However, no commercial nucleic acid vaccine was licensed until the SARS-CoV-2 pandemic hit. As of January 2024, there are two licensed mRNA [7,113] vaccines and one licensed DNA vaccine [114], all against SARS-CoV-2, and many more are being developed, even beyond the infectious disease field. Together with the advantages described for viral vectored vaccines (i.e., fast production and purification, versatility encoding proteins, and pseudotyping), which places them as great vaccine platforms against such a heterogeneous virus as HIV or in pandemic preparedness contexts against emerging viruses (Box 1), DNA and mRNA vaccines do not pose such a strong threat of inducing strong anti-vector responses compared to viral vectors [97,115]. This lower elicitation of anti-vector responses also allows for a longer persistence of the antigen and increases the immunogenicity of the platform. Still, DNA vaccines have sparked some concerns regarding their risk of inducing anti-DNA antibodies and potential integration of DNA into the host cell’s nucleus [116], which are slowly dissipating after their access to the market.

Box 1 **Advantages and disadvantages of DNA and mRNA vaccines.**

Advantages:

- Versatility to encode various forms of antigens (no size limitation for encapsulation):
  ∘ Subunit antigens
  ∘ Membrane-bound antigens
  ∘ Multivalent platforms
- Unique production and purification strategies for all immunogens. No need to optimise downstream purification protocols since the host will be the bioreactor.
- Fast sequence modification: easy to adapt to new threats (emerging pathogens or variants), which accelerates screening speed in preclinical trials.
- Less adverse effects than viral vector vaccines (capillary leak syndrome, vector reactivation, anti-vector responses)

Disadvantages:

- Global production and purification procedures have yet to be easily accessible in developing countries.
- Poor thermostability: requires ultra-freezers for adequate conservation.
- Elevated costs due to intellectual property protection compared to conventional vaccine platforms (viral vectors, inactivated pathogens, subunit proteins).
- Heterogeneous protein production. This limitation is especially relevant for mRNA delivery of proteins in which the therapeutic window is narrower.

#### 4.2.1. Nucleic Acid Vaccines Encoding Viral Particles

DNA vaccines have been tested in clinical trials against HIV [55], SARS-CoV-2 [114], and other diseases too [117]. Regarding HIV, two DNA vaccine products have been tested in efficacy trials (Table 1): in the HVTN505 and in PrEPVacc, as aforementioned. Despite the negative results yielded by these clinical trials, their safety and tolerability have been extensively demonstrated, which can facilitate future attempts with similar platforms. Furthermore, there are a series of DNA vaccine platforms proving their safety profile in phase I/II trials (Table 2). These vaccine candidates in early clinical trials are designed both for the induction of cellular responses against Gag-Pol-Nef proteins (p24CE, HIV.consv, MAG-DNA, or GEO-D03, among others) or humoral responses against Env (DNA-C, DNA Nat-B/ConS, Pennvax, etc.). In these cases, DNA vaccines can also encode cytokines that will act as adjuvants, like IL-12 or GM-CSF [118,119,120]. 

DNA vaccines are often administered using plasmid DNA vectors that contain the gene of interest, a promoter, and a polyadenylation site. One commercially available vector is pVAX1, which follows FDA recommendations for DNA vaccines [121]. The main challenge that nucleic acid administration faces is their limited incorporation into host cells, requiring the use of physical or chemical delivery systems [122]. These systems, such as liposomes or in vivo electroporation, can enhance the transfection efficiency into host cells and improve the desired immune response against the immunogen by mediating an adjuvant effect [123,124].

RNA vaccines gained a significant momentum during the SARS-CoV-2 pandemic due to their successful development and efficacy [125]. This success stemmed from many years of research trying to reduce the immunogenicity of synthetic mRNA, which led to diminishing the rate of rejection and the fast degradation of delivered mRNA [126], and improving their delivery [19]. Before 2020, some attempts at developing HIV RNA vaccines were slowly moving towards clinical trials [18,127], but they were facing some reluctance caused by their novelty [16,128]. However, the success of mRNA vaccines for COVID-19 paved the way for more RNA-based vaccines to enter clinical trials [129]. Still, it is important to remember that regardless of the benefits of the platform used (DNA, mRNA, or viral vectors), if the immunogens that they encode do not elicit potent and protective immune responses, the strategy will still be futile. 

In this sense, the high versatility and robustness of production processes of mRNA vaccine platforms can also prove very useful to accelerate vaccine testing. Since their safety profile is more than demonstrated, smaller, more directed experimental medicine vaccine trials (EMVT) can be performed to answer more directed scientific questions to screen for specific immunogens. This new discovery methods could accelerate vaccine science by testing multiple immunogens in smaller clinical trials, rather than betting on individual products to advance in conventional clinical trials [130]. 

There are currently three mRNA HIV vaccine candidates in phase I clinical trials. These vaccines integrate both the use of mRNA delivery with novel immunogens like the eOD-GT8 oligomer designed to prime CD4bs germline neutralising antibodies (clinicaltrials.gov: NCT05414786 and NCT05001373) or native Env trimers to induce potent neutralising responses (clinicaltrials.gov: NCT05217641). If these trials report successful results regarding their safety and tolerability profiles, and some insights on the immunological profiles they induce, we can expect to see them in combination with Gag-Pol and Nef immunogens to trigger a balanced adaptive immunity in phase II and III clinical trials soon. 

The main challenges that mRNA faces in this context of HIV vaccine development are mainly logistical (Box 1), since the poor thermostable profile and current elevated costs hinder their introduction in developing countries. This stands especially true for Sub-Saharan African countries, where an effective prophylactic vaccine would be most useful to limit transmission. Improvement of the thermostability profile of mRNA [131,132], building better production and storage facilities on site [133], or the development of DNA vaccines as an alternative to mRNA whenever needed, could bypass this limitation. 

#### 4.2.2. Nucleic Acid Vaccines Encoding Virus-like Particles

Another strategy to boost the immunogenicity of subunit vaccines is to express the immunogen on multivalent platforms. Most multivalent platforms use nanotechnology-based strategies such as liposomes, or polymers, but there are also biological approaches that mimic the virus morphology, like virus-like particles (VLPs). These latter strategies can also benefit from nucleic acid vaccine platforms. 

VLPs are as heterogeneous as viruses themselves, and most of them can be used to deliver relevant antigens [134]. However, in a context of an HIV vaccine, it makes sense to develop HIV-based VLPs that mimic the structure of the virus and deliver HIV humoral and cellular immunogens in their native conformation, since size, stability and proper antigen display are key properties that determine the immunogenicity of multivalent platforms [135,136]. 

HIV VLPs are produced by the expression of Gag, HIV’s main structural protein. Similar to HIV virion production, Gag buds on the membrane and VLPs protrude and are excreted, resulting in non-infectious, non-replicative immature VLPs that recapitulate the viral structure [137]. These HIV Gag VLPs can and have been used as immunogens to elicit potent cellular responses by vaccination [138,139], and even tested in humans [140,141,142]. Furthermore, membrane-bound immunogens can be passively incorporated on the VLP surface during budding, resulting in multivalent antigen carriers that express the immunogen in a native-like platform and can trigger both humoral and cellular responses [143]. 

Unfortunately, immunogens are usually incorporated at the surface of HIV Gag VLPs at low densities mirroring Env incorporation at the surface of HIV virions [144], which hinders their ability to induce potent immune responses by reducing antibodies’ avidity. That is why many groups have explored strategies to increase the antigen density at the surface of VLPs by modifying the transmembrane and/or intracellular domains [145,146,147,148], by introducing multimerization tags [149] or by fusing the immunogen with Gag [150,151]. 

A relevant feature of HIV Gag VLPs is that they can be produced in a wide array of in vitro platforms depending on the desired properties [137], but they can also be encoded and produced in vitro upon administration of nucleic acid vaccines [120]. After vaccination with a VLP-encoding nucleic acid vaccine, cells will uptake the DNA or mRNA and start secreting VLPs with immunogens on their surface that can be processed by APCs (Figure 3). Once Env epitopes bind their cognate B cell receptor (BCR), B cells will phagocytose the VLP and present epitopes from that same immunogen via MHC-II. Furthermore, they can also display antigens from proteins present within the particle (like Gag), and increase the chances of getting validated by a wider repertoire of CD4^+^ T helper cells, a phenomenon known as intrastructural help [152,153]. 

There are multiple examples of HIV VLP vaccine candidates formulated as nucleic acid vaccines or administered in heterologous regimens tested in preclinical models (namely mice, macaques, rabbits, or guinea pigs) and early clinical trials [120,148,154,155,156]. Table 3 provides a comprehensive list of some relevant examples of vaccine candidates formulated as nucleic acid encoded VLPs or administered in heterologous regimens and the main immunological results derived from their administration. 

### 4.3. Summary and Future Directions

In conclusion, expanding the heterogeneous armamentarium of vaccines and platforms available for developing preventive vaccination strategies against HIV is crucial to achieve success. As described by many researchers, a successful HIV-1 vaccine will not only depend on developing immunogens and platforms that elicit strong immune responses but also on understanding the synergistic effects of different platforms, immunogens, and adjuvants. 

In this sense, we strongly believe that immunisation strategies with new antigens based on native Env trimers (i.e., SOSIP, NFL, or UFO) displayed as membrane-anchored antigens on cells or, more relevantly, on the surface of multivalent platforms like VLPs, could elicit broadly neutralising humoral responses. Furthermore, VLPs can also contain relevant Gag-, Pol- and/or Nef-based immunogens that can trigger protective cellular responses, achieving a balanced immune response. These multivalent platforms can further benefit from nucleic acid vaccines (mainly DNA and mRNA delivery systems), which facilitates and accelerates upstream and downstream manufacturing and the adaptation of vaccines against highly diverse viruses like HIV or influenza viruses, or against emerging viruses or viral variants of concern, that can pose a threat for humans. 

Considering that both nucleic acid delivery systems, as well as VLPs have proven safe in clinical trials, we deem feasible that they could be successfully used in combination as vaccine platforms to display key immunogens in a future attempt to develop a prophylactic HIV vaccine, either alone or in combination with other immunogens in heterologous prime/boost systems. These strategies could provide a fresh push to pursue a new wave of clinical trials for the prevention of HIV infections, and even beyond this field. 

## Figures and Tables

**Table 1:** HIV vaccine efficacy trials performed up to date.

Name	Code	Phase	Immunogen	Formulation					Main Finding	Study Group	Status	Year	Location
				DNA	Poxvirus	AVs	Protein	Adjuvant					
Vaccines that aimed at eliciting neutralising humoral responses													
Vax 003	NCT00006327	III	Bivalent Clade B&E gp120	-	-	-	AIDSVAX B/E	Alum	No protection	IDU	Complete	2003	Thai
Vax 004	NCT00002441	III	Bivalent Clade B&B gp120	-	-	-	AIDSVAX B/B	Alum	No protection. Low Tier1 nAbs	MSM and high risk women	Complete	2004	USA Netherlands
Vaccines that aimed at inducing protective cellular responses													
STEP HVTN502	NCT00095576	IIb	Clade B *gag*, *pol* & *nef*	-	-	Ad5	-	-	Anti-vector Abs may increase risk of infection	High risk population	Stopped	2007	America Australia
Phambil HVTN503	NCT00413725	IIb	Clade B *gag*, *pol* & *nef*	-	-	Ad5	-	-		South-African population	Stopped	2007	South-Africa
Vaccines that aimed at generating balanced adaptive immune responses													
RV144	NCT00223080	III	Clade B *gag-pro*, *env AE* Clade B&E gp120	-	ALVAC	-	AIDSVAX B/E	Alum	31.2% protection	Thai population	Complete	2009	Thai
HVTN505	NCT00865566	IIb	Clade B *gag*, *pol* & *nef* Clades A, B, C *env*	6 p.	-	rAd5	-	-	No protection. Anti-gp41 nnAbs	MSM and transgenders	Stopped	2013	USA
UHAMBO HVTN 702	NCT02968849	III	Clade B/C *gag-pro* & *env* Clade C gp120	-	ALVAC	-	Cgp120 C	MF59	No protection demonstrated	South-African population	Stopped	2020	South-Africa
IMBOKODO HVTN 705	NCT03060629	IIb	Mosaic gag, pol & *env* Clade C Env trimer	-	-	Ad26	Clade C gp140	Alum	No significant protection (25.2%)	Women at risk	Stopped	2021	Sub-saharan Africa
MOSAICO HVTN 706	NCT03964415	III	Mosaic *gag*, *pol* & *env* Clade C + Mosaic gp120	-	-	Ad26	TV1.Cgp120 1086.Cgp120	Alum	No protection demonstrated	MSM and transgenders	Stopped	2023	Europe America
PrEPVacc	NCT04066881	IIb	Clade C *gag*, *pol*, *env*, *nef* Env E *Gag*-*pol* Clade A Clade B&E/Clade C Env	3 p.	MVA	-	CN54gp140 AIDSVAX B/E	MPLA	No protection demonstrated	High risk population	Stopped	2023	Uganda

Words in italics indicate viral genes. p.: plasmids; IDU: intravenous drug users; nAbs: neutralising antibodies; MSM: men who have sex with men; Ad5: adenovirus 5; Abs: antibodies; nnAbs: non-neutralising antibodies; MPLA: monophosphorylipid A.

**Table 2:** DNA and mRNA HIV vaccines tested in the last 10 years.

Name	Immunogen	Combined Strategy	No.	Adjuvants	Delivery	RoA	Phase	Main Finding	Date	
DNA vaccines										
HIVIS-DNA	Subtypes A, B, C *env*, *gag* & B *rt/rev*	MVA	+	TLR4 agonist	EP	ID	I	Ongoing	-	NCT04301154
Env-C DNA	Subtype C *env*	Subunit protein	1	Alum/ALF43	-	IM	I	Ongoing	-	NCT04826094
PDPHV201401	Subtype A, B, C, A/E *env* & C *gag*	Subunit protein	5	GLA-SE	-	IM	I	Ongoing	-	NCT04927585
DNA-HIV-PT123	Subtype C *env*, *gag* & B/C *pol-nef*	Subunit/MVA	3	Alum	-	IM	IIb	Safe, stopped (no efficacy)	2023	NCT04066881
Env/Gag DNA vaccine	Subtypes A, B, C, A/E *env* & C *gag*	Subunit protein	5	-	-	IM	I	Safe, broad and potent IR	2021	NCT03409276
p24CE1/2 & p55_DNA	Conserved elements of p24 & full p55	-	2	IL12	EP (Ichor)	IM	I	Safe, EP induced higher IR	2020	NCT03181789
DNA Nat-B/ Con-S/mosaic	Clade B, Consensus M and Mosaic *env*	MVA	3	-	-	IM	I	N.Y.R	2020	NCT02296541
nef/tat/vif, env pDNA	N.D.	MVA	1	-	EP		I	Completed	2019	NCT02654080
HIV DNA-C CN54ENV	Subtype C *env*	Subunit protein	1	-	EP (Ichor)	IM/ID	I	-	2019	NCT02654080
Pennvax-GP Pennvax-B	Cons. A&C *env*	-	1	IL12	EP (Cellectra)	ID/IM	I	Robust Ab and cellular IR	2018	NCT02431767
GEO-D03 DNA	Subtype B *env*, *gag*, *pol*	MVA	1	GM-CSF	-	IM	I	Safe, good for priming	2017	NCT01571960
pSG2.HIVconsv	HIV.consv construct	ChAdV or MVA	1	-	-	IM	I	Safe, high CTL	2017	NCT01151319
MAG-pDNA	Subtype B *env*, *gag*, *pol*, *nef*, *tat* & *vif*	rVSV	2	IL-12	EP (Ichor)	IM	I	Safe, primes CTL	2016	NCT01578889
mRNA vaccines										
V3G CH848 mRNA-Tr2	Native-like *env* trimer	Nanoparticle	1	Alum	Liposome	IM	I	Recruiting	-	NCT05903339
BG505 trimer	Native-like *env* trimer	-	1		Liposome	IM	I	Ongoing	-	NCT05217641
mRN1644	eOD-GT8 & Core-g28v2	mRNA	1	-	Liposome	IM	I	Ongoing	-	NCT05001373

Words in italics indicate viral genes. No.: number of plasmids; RoA: route of administration; TLR: toll-like receptor; ID: intradermal; IM: intramuscular; MVA: modified vaccinia Ankara; EP: electroporation; IR: immune response; N.Y.R.: not yet recruiting; N.D.: not determined; CTL: cytotoxic T lymphocytes.

**Table 3:** Immunogenicity studies of Gag-based VLPs as nucleic acid vaccine candidates for HIV.

Surface Immunogen	Adjuvant	Aim	Production Platform	Model	Immunisation Regimen	RoA	Major Findings	Ref.
Nucleic acid HIV-1 Gag VLPs								
Subtype B Env	-	Test DNA/MVA vaccine forming VLPs	In vivo	Macaques	Heterologous (DMM)	IM	Superior Ab response in VLP constructs	[154]
CRF02_AG Env	-	Test DNA/MVA CRF02_AG in mVLPs vs VLPs	In vivo	Macaques	Heterologous (DDDM)	IM	Better cellular and humoral response in mVLP	[155]
Clade B Env	GM-CSF	Safety & immunogenicity	In vivo	Humans	MVA	IM	Safe, good for priming	[120]
Clade C gp145	-	Compare combine Gag-Pol-Env vs single MVAs	In vivo	BALB/c	MVA	IM	Superior Ab & CTL in the combined MVA	[156]
Subtype C gp160	-	Increase Env density at VLP surface	In vivo	Rabbit	Heterologous (DDMMPP)	IM	No high-density Env in VLPs, Tier 2 autologus nAb	[148]
Combined Nucleic acid VLPs and Gag-VLPs								
Subtype B Env	QS21	Assess immunogenicity	Vero cells In vivo	Macaques	Heterologous (D/V/A)	IM	Autologous nAbs and CTL	[157]
Subtype B Env	-	Evaluate VLP as a boost for D/Fpox	Vero cells In vivo	Rabbits	Heterologous (D/Fpox/V)	ID	VLP boosting elicits potent Ab	[158]
Subtype B Env	-	Compare heterologous immunisation	Vero cells In vivo	BALB/c mice	Heterologous (3xD/Fpox + VV)	IM/SC	nAbs & CTLs control env-tumour cells	[159]
Clade A Gp120	Eurocine	Compare mucosal immunisation	Insect cells In vivo	BALB/c mice	Homologous (VV) Heterologous (DV)	IN	Superior Ab & CTL with heterologous vaccination	[160]
Clade B gp160 and Gp120	-	Check S2 cell VLP platform	S2 cells In vivo	BALB/c	Heterologous (DDVVV)	SC	Antibody induction and modest neutralisation.	[161]
Gp41 variants	-	Induce anti-MPER bNAbs	HEK293T cells In vivo	BALB/c	Heterologous (DDDVVV)	SC	Trimeric MPER increases Ab responses	[162]
Clade B gp140	-	Intrastructural help testing	HEK293T cells In vivo	C57BL/6J	Heterologous (Ad or DNA + VLP)	Footpad	Gag boosts Env via intrastructural help	[163]
Clade B Env	Alum or CpG	To test mixing PrEP and vaccine	COS cells In vivo	Rhesus macaque	Heterologous (DDVVV) + PrEP	IM/IN	Combining PrEP + vaccine confers higher protection	[164]
Truncated gp41	Carbopol974	Induce anti-MPER bNAbs	HEK293F cells In vivo	Rabbit (NZW)	Heterologous (DDVV)	IM	Anti-MPER Ab and low nAbs by gp41-DDVV	[165]
Clade B and C Env	-	Test CT on VLP immunogenicity	Insect cells In vivo	Guinea pig	Heterologous (DDVV)	IM	Modified Env CT enhances VLP immunogenicity	[166]
SIV239 Env	CD40L, MPLA, R848	Induce protective IR in rhesus	-	Rhesus macaque	Heterologous (DDMMV)	Hock	Partial protection in DDMMV group	[167]
Subtype B gp140	IL12, IL28	Induce balanced response by ISH	293T cells In vivo	BALB/c mice	Homologous (DD) Heterologous (DVV)	IM	Higher Ab & CTL by heterologous ISH-based vaccine	[168]
Subtype B gp120	c-di-GMP	Test in vivo EP in an heterologous regimen	Insect cells In vivo	BALB/c mice	Heterologous (DDVV)	SC	Th1-like response in DDVV w/nAb & CTL	[169]
Clade B Env	Adjuplex	Induce bNAbs	HEK293F cells In vivo	Rabbit	Heterologous (DDVVV)	-	Enhanced Ab response by 4E10-selected VLPs	[170]
Subtype B Env	R848	Test PIV vector as a prime	FreeStyle 293 In vivo	Macaques	Heterologous (PIV/VLP)	IN/IM	Potent IR, previous anti-PIV exposition no major effect	[171]
Subtype B Env	Adjuplex (prot)	mRNA delivery of Gag VLPs expressing Env	HEK293F cells In vivo	Mice & macaques	Heterologous (mRNA, clades or proteins)	IM	79% cross-clade risk reduction in macaques	[172]
Subtype B SOSIP	Adjuplex	Compare DDPPP vs DDVVV w/ SOSIP Env	FreeStyle 293 In vivo	Rabbits	Heterologous (DDVVV)	-	Strong Ab response DDPPP and DDVVV (lower dose)	[173]
Truncated gp41	-	Genereate high-density VLPs to induce strong Abs	In vivo Expi293 cells	C57BL/6J	Heterologous (DDVV)	SC	ADCC responses & control gp41-tumour cells	[150]
Clade B gp140	-	To induce mucosal IR w/MLV-Gag VLP	In vivo HEK293T cells	BALB/c	Heterologous (DV or DP)	Intravag.	Induction of systemic and mucosal immunity	[174]

RoA: route of administration; IM: intramuscular; ID: intradermal; SC: subcutaneous; IN: intranasal; intravag.: intravaginal; M or MVA: modified vaccinia Ankara; D or DNA: deoxyribonucleic acid; V or VLP: virus-like particles; mVLPs: mature VLPs; CTL: cytotoxic T lymphocyte; Ab: antibodies; P: protein; A or Ad: adenovirus; Fpox: fowlpox; nAbs: neutralising antibodies; MPER: membrane proximal external region; bNabs: broadly neutralising antibodies; PrEP: pre-exposure prophylaxis; CT: C-terminus; IR: immune response; ISH: intrastructural help. ADCC: antibody-dependent cellular cytotoxicity; MLV: murine leukemia virus; PIV: parainfluenza virus.
